# Endogenous Brain Pericytes Are Widely Activated and Contribute to Mouse Glioma Microvasculature

**DOI:** 10.1371/journal.pone.0123553

**Published:** 2015-04-13

**Authors:** Andreas Svensson, Ilknur Özen, Guillem Genové, Gesine Paul, Johan Bengzon

**Affiliations:** 1 Lund Stem Cell Center, BMC B10, Skåne University Hospital, Lund, Sweden; 2 Division of Neurosurgery, Department of Clinical Sciences, Skåne University Hospital, Lund, Sweden; 3 Translational Neurology Group, Division of Neurology, Department of Clinical Sciences, Wallenberg Neuroscience Center, Lund University and Skåne University Hospital, Lund, Sweden; 4 Division of Vascular Biology, Department of Medical Biochemistry and Biophysics, Karolinska Institute, Stockholm, Sweden; University of Michigan School of Medicine, UNITED STATES

## Abstract

Glioblastoma multiforme (GBM) is the most common brain tumor in adults. It presents an extremely challenging clinical problem, and treatment very frequently fails due to the infiltrative growth, facilitated by extensive angiogenesis and neovascularization. Pericytes constitute an important part of the GBM microvasculature. The contribution of endogenous brain pericytes to the tumor vasculature in GBM is, however, unclear. In this study, we determine the site of activation and the extent of contribution of endogenous brain pericytes to the GBM vasculature. GL261 mouse glioma was orthotopically implanted in mice expressing green fluorescent protein (GFP) under the pericyte marker regulator of G protein signaling 5 (RGS5). Host pericytes were not only activated within the glioma, but also in cortical areas overlying the tumor, the ipsilateral subventricular zone and within the hemisphere contralateral to the tumor. The host-derived activated pericytes that infiltrated the glioma were mainly localized to the tumor vessel wall. Infiltrating GFP positive pericytes co-expressed the pericyte markers platelet-derived growth factor receptor-β (PDGFR-β) and neuron-glial antigen 2. Interestingly, more than half of all PDGFR-β positive pericytes within the tumor were contributed by the host brain. We did not find any evidence that RGS5 positive pericytes adopt another phenotype within glioma in this paradigm. We conclude that endogenous pericytes become activated in widespread areas of the brain in response to an orthotopic mouse glioma. Host pericytes are recruited into the tumor and constitute a major part of the tumor pericyte population.

## Introduction

Glioblastoma multiforme (GBM) is the most common and aggressive primary brain tumor in adults, with a median survival of only 14.6 months even when all available treatment is given [1]. One major reason for this poor survival is the rapid and infiltrative growth pattern of the tumor, facilitated by extensive angiogenesis and neovascularization [2]. An important cellular component of the GBM vasculature is the pericytes. Tumor pericytes mediate immunosuppression [3] and promote endothelial cell survival [4,5], thus facilitating tumor growth. Pericytes aligning glioma vessels are often abnormal and scarcer compared to pericytes on normal vessels [6,7], resulting in a dysfunctional vasculature and blood-brain barrier.

The source of pericytes in GBM remains controversial. A proportion of pericytes in GBMs are generated from tumor stem cells residing within the GBM itself [8] or recruited from the bone marrow [9]. However, whether brain-derived pericytes contribute to the tumor vasculature is not known.

Here we investigate the contribution of normal brain pericytes to GBM vasculature using an orthotopic mouse glioma model. We have recently shown [10] that pericytes in the human brain resemble perivascular multipotent mesenchymal stromal cells that share characteristics of both pericytes and mesenchymal stromal cells [11]. Recent observations in several tissues indicate that pericytes are versatile and have the ability to respond to environmental stimuli such as stroke [12]. Furthermore, mesenchymal stromal cells, similar to pericytes, have a strong tumor tropism and migratory capabilities, and integrate with the tumor vessels as pericyte-like cells upon intratumoral implantation [13].

In the present study we used mice where green fluorescent protein (GFP) is expressed under the pericyte-specific promoter regulator of G protein signaling 5 (RGS5) [14,15] and thus labels host-derived pericytes. We implanted GL261 mouse glioma cells [16,17] into these mice and show that endogenous pericytes are activated in widespread areas of the brain, recruited into established intracerebral GL261 gliomas and integrate with the tumor vessels. Quantification revealed that more than half of all platelet-derived growth factor receptor-β (PDGFR-β) positive pericytes within the glioma are host brain-derived.

## Materials and Methods

### Ethics Statement

All animal procedures were approved by the Committee of Animal Ethics in Lund-Malmö, Sweden (permit number: M259-12).

### Cell Line and Culture

The GL261 mouse glioma cell line [16,17], syngenic to the C57BL/6 mouse strain, was a kind gift from Dr. Géza Sáfrány, Hungary. The cells were cultured in R10 medium (RPMI 1640 medium supplemented with 300 μg/ml l-glutamine, 1 mM sodium pyruvate, 10 mM HEPES, 50 μg/ml gentamicin (Life Technologies, Carlsbad, CA, USA) and 10% fetal bovine serum (FBS) (Biochrom AG, Berlin, Germany)) at 37°C in a humidified atmosphere containing 5% CO_2_. For *in vivo* inoculation, cells were resuspended in R0 medium (R10 medium without gentamicin and FBS).

### GL261 Tumor Cell Inoculation *in vivo*

We used the reporter rgs5^GFP/+^ mice, a knock-out/knock-in C57BL/6 mouse line expressing GFP under the pericyte-specific RGS5 promoter [15]. Heterozygote rgs5^GFP/+^ females between 7–17 weeks of age, or 14 weeks old wild-type C57BL/6 females, were anaesthetized with isoflurane (Forene, Abbott Scandinavia AB, Solna, Sweden) and placed in a stereotaxic frame (David Kopf Instruments, Tujunga, CA, USA). They received local anaesthetic by subcutaneous injection of 0.025 ml Marcain with adrenaline (2.5 mg/ml bupivacaine, 5 μg/ml epinephrine, AstraZeneca AB, Södertälje, Sweden) on the skull. A hole was drilled and 5000 GL261 tumor cells in 5 μl R0 medium were injected at 1 μl/min into the caudate nucleus using a 10 μl Hamilton syringe (Hamilton Bonaduz AG, Bonaduz, Switzerland) at the following coordinates: 1.5 mm lateral and 1.0 mm anterior of bregma, 2.75 mm ventral of the skull bone. After injection, the needle was left in the brain for 5 minutes before it was slowly retracted. The hole in the skull was sealed with bone wax. At day 19 after tumor inoculation, animals were cardially perfused with 0.9% NaCl solution (Merck KGaA, Darmstadt, Germany) followed by 4% paraformaldehyde (PFA, Electron Microscopy Sciences, Hatfield, PA, USA). Brains were removed and postfixed in 4% PFA at 4°C overnight before being transferred to 30% sucrose solution (Merck KGaA). The brains were sectioned in 40 μm thick coronal sections with a Leica SM200 R sliding microtome (Leica Biosystems Nussloch GmbH, Nussloch, Germany) and stored at -20°C in anti-freeze solution (30% etylen glycol and 30% glycerol (both from VWR International, Radnor, PA, USA) in 0.012 M NaH_2_PO_4_·H_2_O and 0.031 M Na_2_HPO_4_·2H_2_O (both from Sigma-Aldrich, Stockholm, Sweden)) for subsequent histological staining.

### Immunofluorescence

Free-floating sections were washed three times in phosphate-buffered saline (PBS, Life Technologies) and Fc receptors were blocked with Innovex Fc Receptor Blocker (Innovex Biosciences Inc., Richmond, CA, USA) in accordance to the manufacturer’s instructions. Sections were blocked with 5% normal goat serum (NGS, Jackson ImmunoResearch Europe Ltd., Suffolk, United Kingdom) and 0.5% Triton X-100 (Sigma-Aldrich) in PBS (PBTX) and then incubated with chicken anti-GFP antibody (1 μg/ml, Abcam, Cambridge, United Kingdom) in 0.5% PBTX supplemented with 3% NGS at room temperature overnight. The next day, the sections were washed three times in PBS and subsequently incubated with biotinylated goat anti-chicken antibody (6 μg/ml, Vector Laboratories Ltd., Peterborough, United Kingdom) in 0.5% PBTX supplemented with 3% NGS at room temperature for 2 hours. The sections where then washed three times in PBS and incubated with Alexa Fluor 594-conjugated streptavidin (7.2 μg/ml, Jackson ImmunoResearch Europe Ltd.) in 0.5% PBTX supplemented with 3% NGS at 4°C for 2 hours.

All sections were washed three times with PBS. Sections stained for CD13 and CD31 were blocked with 5% normal donkey serum (NDS, Jackson ImmunoResearch Europe Ltd.) in 0.5% PBTX. Sections stained for PDGFR-β and glucose transporter 1 (GLUT1) were incubated in 10 mM citrate buffer (10 mM trisodium citrate dehydrate supplemented with 0.05% Tween 20 (both from Sigma-Aldrich), pH 6.0) at 80°C for 30 minutes for antigen retrieval and then washed three times in PBS. Sections were incubated with either rabbit anti-laminin antibody (1.2 μg/ml, Abcam), rabbit anti-Ki67 antibody (2.5 μg/ml, Abcam), rabbit anti-PDGFR-β antibody (diluted 1:200, Cell Signaling Technology Europe, B.V., Leiden, The Netherlands), rabbit anti-GLUT1 antibody (5 μg/ml, Abcam), rabbit anti-vascular endothelial growth factor receptor 2 (VEGF-R) antibody (diluted 1:200, Cell Signaling Technology Europe), rabbit anti-α-smooth muscle actin (α-SMA) antibody (2 μg/ml, Abcam), rat anti-CD13 antibody (2 μg/ml, AbD Serotec, Kidlington, United Kingdom), rabbit anti-ionized calcium binding adapter molecule 1 (Iba1) antibody (0.25 μg/ml, Wako Chemicals GmbH, Neuss, Germany), rabbit anti-S100 calcium binding protein B (S100B) antibody (diluted 1:500, Abcam), rabbit anti-neuron-glial antigen 2 (NG2) antibody (2 μg/ml, Merck Millipore, Billerica, MA, USA) or rat anti-CD31 antibody (0.04 μg/ml, BD Biosciences, Heidelberg, Germany) in 0.5% PBTX with either 3% NDS or 3% NGS overnight. The anti-laminin, anti-GLUT1, anti-CD13 and anti-CD31 antibodies were incubated at 4°C and the rest at room temperature. After incubation, the sections were washed three times in PBS and then incubated with secondary Alexa Fluor 647 goat anti-rabbit, Alexa Fluor 647 donkey anti-rat or DyLight 649 donkey anti-mouse antibody (3 μg/ml, all from Jackson ImmunoResearch Europe Ltd.) in 0.5% PBTX at room temperature for 2 hours. After incubation, the sections were washed three times in PBS and then stained with Hoechst 33342 (8.1 μM, Life Technologies) for 10 minutes. The sections were washed three more times and then mounted on SuperFrost Plus glasses (Thermo Fisher Scientific Inc., Waltham, MA, USA) with DABCO (Sigma-Aldrich) and coverslipped.

Brains from five tumor-bearing mice were used for the GLUT1 analysis and brains from three tumor-bearing mice were used for the analysis of the other markers.

### DAB Staining

Free-floating sections were washed three times in PBS and then quenched in PBS supplemented with 3% H_2_O_2_ (Merck KGaA) and 10% methanol (J.T.Baker, Avantor Performance Materials B.V., Deventer, The Netherlands) for 15 minutes. Sections were blocked with 5% NGS and 1% Tween 20 in PBS and then incubated with chicken anti-GFP antibody (2 μg/ml) in PBS supplemented with 3% NGS at room temperature overnight. The next day, the sections were washed three times in PBS and subsequently incubated with biotinylated goat anti-chicken antibody (6 μg/ml) in PBS supplemented with 3% NGS at room temperature for 2 hours. The sections where then washed three times in PBS and the biotinylated antibody was visualized with the VECTASTAIN Elite ABC Kit and the DAB Peroxidase Substrate Kit (both from Vector Laboratories Ltd.) in accordance to the manufacturer’s instructions. The sections were washed three more times and then mounted on gelatin-coated glasses (Thermo Fisher Scientific Inc.), left to dry overnight and coverslipped using DPX mounting medium (Sigma-Aldrich).

Brains from three tumor-bearing mice were used for the DAB analysis and tumor-free brains from three mice were used as control.

### Confocal Microscopy

The immunofluorescent tissue sections were analyzed on a Zeiss LSM 780 confocal microscope (Carl Zeiss Microscopy GmbH, Jena, Germany). Hoechst 33342 was excited with the 405 nm laser and light was collected between 410–476 nm. Alexa Fluor 594 was excited with the 561 nm laser and light was collected between 588–642 nm. Alexa Fluor 647 and DyLight 649 were excited with the 633 nm laser and light was collected between 654–755 nm. To determine the amount of autofluorescence from the 561 nm laser, the sections were also excited with the 488 nm laser and light was collected between 491–571 nm or 589–643 nm. No specific staining was visualized with these settings, but the autofluorescent parts could be defined and subtracted from the real Alexa Fluor 594 staining.

### Digital Image Processing

The immunofluorescence images of Alexa Fluor 594 were digitally enhanced by removing autofluorescent elements and noise in Adobe Photoshop CS5.1 (Adobe Systems Inc., San Jose, CA, USA). Autofluorescence was removed using two separate single channel images. The first image was excited with the 561 nm laser and contained true antibody-mediated staining as well as autofluorescence. The second image was captured with the 488 nm laser, which only slightly excited Alexa Fluor 594, and hence only contained the non-specific autofluorescence. The images were overlayed in Adobe Photoshop and the exposures were adjusted to match. Each pixel value of the 561 nm image was then compared to the corresponding pixel value of the 488 nm image, and the difference was calculated. A new single channel image was created, where each pixel value was the difference between the corresponding pixel values of the original images. This was done using the *Difference* blend mode. The result was an image where elements present in both original images, i.e. autofluorescence, were removed while elements only present in one of the images, i.e. antibody-mediated staining, were preserved. Finally, the *Dust & Scratches* filter was applied to reduce image noise.

### Stereology

Imaging and quantification of the DAB stained sections was conducted with an Olympus BX53 system microscope (Olympus, Shinjuku, Tokyo, Japan). Each brain was divided into two regions of interest (ROIs); one outside the tumor and one in the corresponding location in the contralateral hemisphere. Three images at 20x magnification, resulting in a total area of 0.277 mm^2^, were taken from each ROI and the GFP positive cells were manually counted in each image.

To quantify the number of cells positive for GFP, PDGFR-β, NG2, CD13, α-SMA and Ki67, confocal z-stacks from three different tumors were taken at 20x magnification. Each z-stack consisted of 10 sequential, 1.14 to 1.77 μm thick, optical sections showing the expression of GFP and one additional marker. The area counted in each tumor was 0.181 mm^2^. To count the number of cells expressing each marker, all optical sections were analyzed in Adobe Photoshop CS5.1 one by one. The cells, visualized by fluorescent staining, were manually counted. To avoid counting the same cell several times, each cell was marked in a transparent layer that was moved between all the optical sections within the z-stack. At last, the number of cells expressing GFP alone, one of the other markers alone, or co-expressing GFP with one of the other markers was determined for each z-stack.

### Statistics

The cell counting analysis was performed using ANOVA, where p<0.05 was considered statistically significant.

## Results

### Pericytes are Activated in Widespread Areas of the Brain in Response to Local Glioma Growth

**Fig 1:**
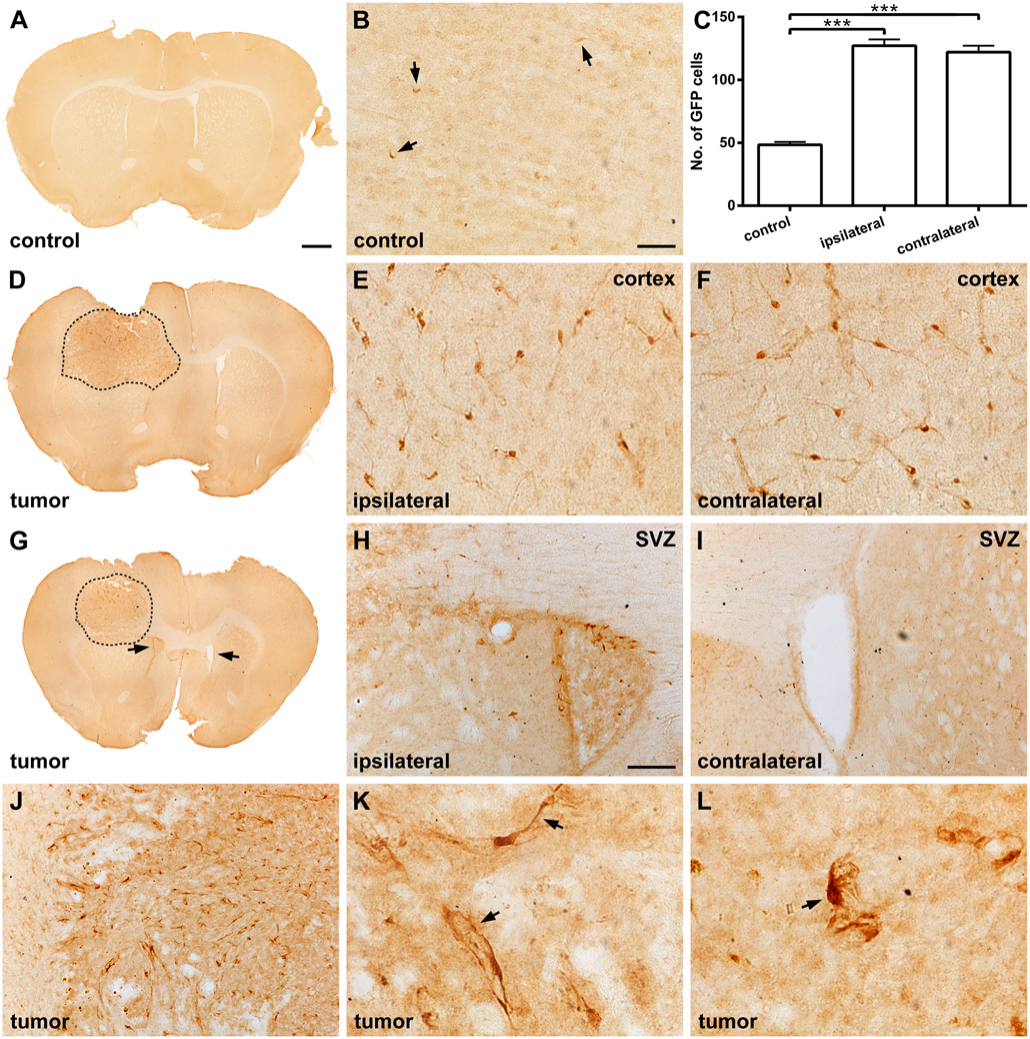
Pericytes are activated around glioma. (A) Low magnification photomicrograph of a normal rgs5^GFP/+^ mouse brain, scale bar is 500 μm. (B) Under normal conditions, quiescent GFP positive pericytes showed a flat morphology with a small cell body (arrows), scale bar is 20 μm. (C) However, in response to a GL261 glioma, the number of GFP positive pericytes within the cerebral cortex was significantly increased, both in the ipsilateral and contralateral hemisphere, compared to a normal mouse brain without tumor (n = 3, mean ± SEM, ***, p<0.001, ANOVA). (D) Low magnification photomicrograph of a representative GL261 tumor (dashed) in the rgs5^GFP/+^ mouse brain, scale bar as in A. Pericytes showing a morphology consistent with activated pericytes were found in the cerebral cortex both in the (E) ipsilateral and (F) contralateral hemisphere, scale bars as in B. (G) Low magnification photomicrograph of a representative GL261 tumor (dashed) in the rgs5^GFP/+^ mouse brain showing the SVZ (arrows), scale bar as in A. Activated pericytes are present (H) in the SVZ ipsilateral to the tumor but not (I) in the SVZ contralateral to the tumor, scale bar is 50 μm. (J) The morphology of the GFP positive pericytes inside the tumor was different compared to the pericytes in the cortex, with either (K) a flattened cell body with elongated processes (arrows) or (L) a prominent cell body with tuft-like processes (arrow). Scale bar in J as in H and scale bars in K-L as in B.

Under normal conditions, GFP positive pericytes show a flat morphology with a small cell body indicating a quiescent state (Fig 1A and 1B). However, under pathological conditions they can become activated and show a more prominent cell body [18]. In brains containing glioma, the number of activated GFP positive pericytes within the cerebral cortex, adjacent to and overlying the tumor, was significantly increased compared to the corresponding region of rgs5^GFP/+^ mice not harboring tumor (mean: 127 ± 4.97 and 48.5 ± 2.28, respectively; p<0.001) (Fig 1C–1E). The number of activated GFP positive pericytes was also significantly increased within the contralateral hemisphere of tumor-bearing rgs5^GFP/+^ mice, indicating a widespread activation of perivascular cells (Fig 1C, 1F). Similar to cerebral cortex, activated GFP positive pericytes were consistently found in the rostral subventricular zone (SVZ) of the lateral ventricle ipsilateral to the glioma as compared to the SVZ contralateral to the tumor (Fig 1G–1I). The morphology of the GFP positive pericytes in the host cortex was consistent with activated pericytes (Fig 1E and 1F), while the GFP positive pericytes within the tumor were found to have different morphological profiles (Fig 1J–1L). They had either a flattened cell body with elongated processes (Fig 1K) or a prominent cell body with retracted finger-like projections (Fig 1L). Both cell types were found throughout the whole tumor with no area-specific distribution pattern. However, the flattened morphology was mainly localized close to the tumor border whereas cells with a large cell body and prominent projections were evenly distributed within the tumor.

### Activated Pericytes are Associated with Laminin Positive Tumor Microsatellites

**Fig 2:**
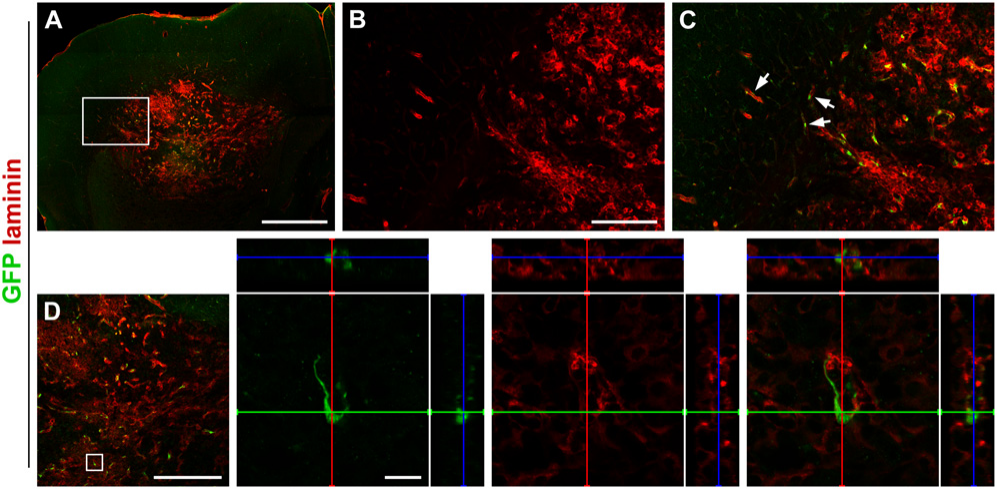
Pericytes are associated with laminin-expressing tumor satellites. (A) Overview image of GL261 tumor expressing high levels of laminin, scale bar is 1000 μm. (B) Higher magnification of laminin expression around the tumor border, scale bar is 200 μm. (C) GFP positive pericytes localize close to laminin-expressing tumor microsatellites outside the tumor (arrows), scale bar as in B. (D) However, they do not express laminin themselves. Scale bar is 500 μm in the low magnification image and 20 μm in the high magnification images.

Recent studies of glioma have shown that laminins are important for glioma cell invasion and growth [19]. Given these findings, we next examined the expression of laminin in GL261 tumors in rgs5^GFP/+^ mice 19 days after tumor inoculation and found laminin to be highly expressed by these tumors (Fig 2A and 2B). Laminin positive glioma microsatellites were found at the glioma/brain interface at some distance from the main tumor bulk. Migrating GFP positive pericytes were located close to these laminin positive satellites, not only adjacent to the tumor but also at a distance to its margin (Fig 2C). The GFP positive pericytes did not co-express laminin (Fig 2D).

### Activated Pericytes are Attracted to Hypoxic Tumor Regions

**Fig 3:**
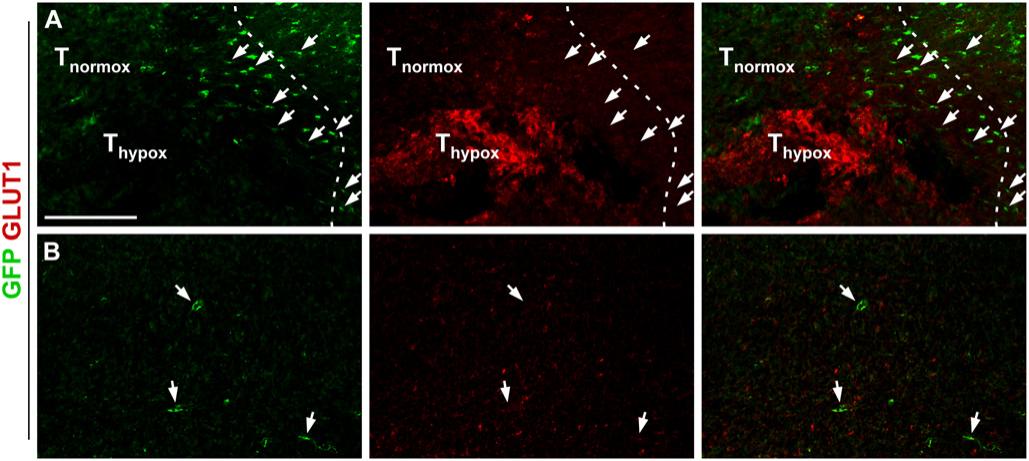
GFP positive pericytes are found preferably within hypoxic regions of glioma. (A) GFP positive cells (arrows) are attracted more numerous within the penumbra zone around GLUT1 positive hypoxic regions close to the GL261 tumor border (dashed), scale bar is 200 μm. Normoxic and hypoxic parts of the tumor are marked with T_normox_ and T_hypox_, respectively. (B) Few GFP positive pericytes (arrows) are found at normoxic regions within the tumor, scale bar as in A.

Hypoxic regions are a well-known characteristic of malignant gliomas. Next, we investigated whether the recruitment of activated pericytes was related to hypoxia by staining for GLUT1, a transport protein upregulated at hypoxic conditions due to the increased need for glucose [20]. Although GFP positive pericytes were found at both normoxic and hypoxic regions of the tumor, they were clearly more numerous around areas of GLUT1 immunoreactivity. The GLUT1 positive tumor areas were mainly localized in the periphery of the tumor. Close to these hypoxic areas at the interface between brain and tumor, GFP positive pericytes appeared to form a stream of migrating cells from the brain into the penumbra zone around the GLUT1 positive areas (Fig 3A and 3B). No GLUT1 positivity was observed within or near the SVZ.

### The Majority of the PDGFR-β Positive Pericytes within the Tumor are Host-Derived

**Fig 4:**
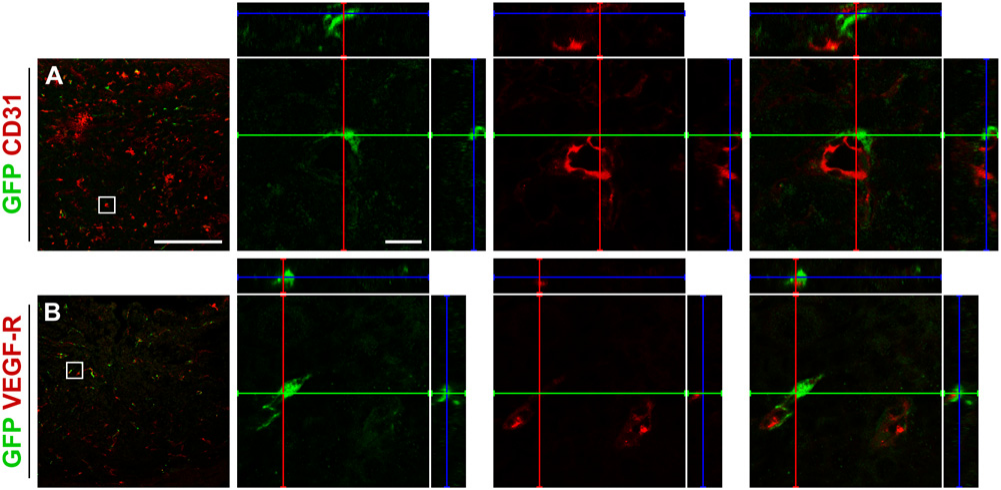
Pericytes within the tumor localize adjacent to vessels. The GFP positive cells within GL261 tumors are localized near cells expressing (A) CD31 and (B) VEGF-R, but do not express the markers themselves. Scale bar is 500 μm in the low magnification images and 20 μm in the high magnification images.

Glioma vasculature consists of dilated and tortuous vessels expressing markers such as CD31 (Fig 4A) and VEGF-R (Fig 4B) [21,22]. Approximately three quarters of all VEGF-R expressing tumor vessels in the GL261 glioma in the present study were covered by GFP positive pericytes.

**Fig 5:**
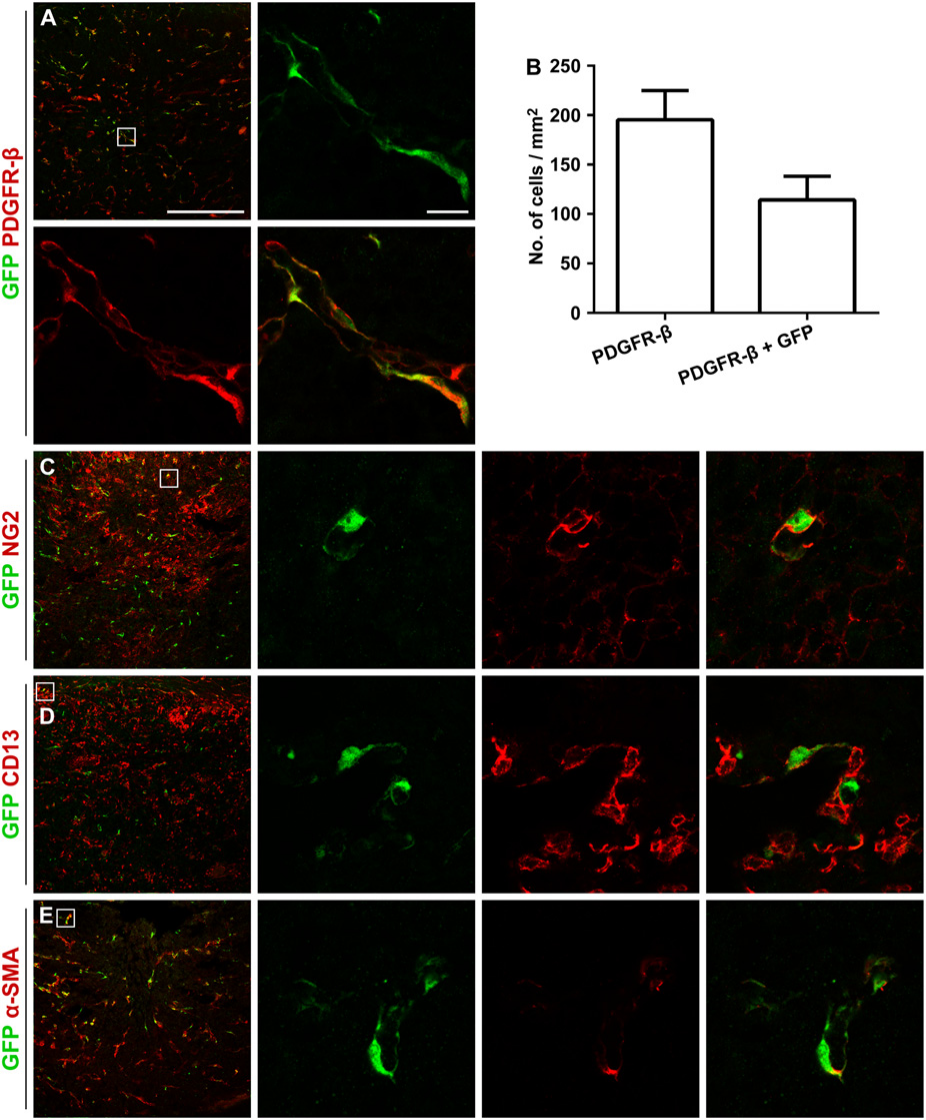
GFP positive cells express pericyte markers. (A) All GFP positive cells within the GL261 tumor are clearly positive for PDGFR-β. (B) Out of all PDGFR-β positive cells within the tumor, 57 ± 6.6% are host-derived GFP positive cells (n = 3, mean ± SEM). (C) A proportion of the cells are positive for the activation marker NG2. The majority of the GFP positive cells lack expression of the mesenchymal stromal cell marker CD13. (D) GFP positive cell at the tumor border expressing CD13. (E) The majority of the cells weakly express the pericyte marker α-SMA. Scale bar is 500 μm in the low magnification images and 20 μm in the high magnification images.

**Fig 6:**
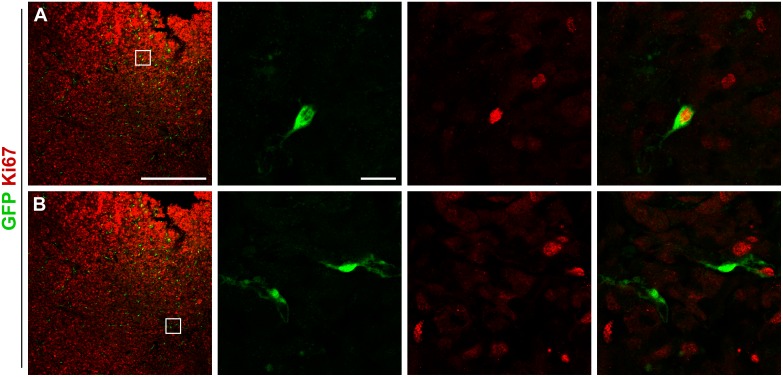
A proportion of the pericytes proliferate intratumorally. (A) A subset of the GFP positive cells within GL261 tumors express the proliferation marker Ki67, (B) whereas a majority of the cells do not. Scale bar is 500 μm in the low magnification images and 20 μm in the high magnification images.

Interestingly, out of all PDGFR-β positive cells associated with microvessel walls within the GL261 glioma, 57 ± 6.6% co-labeled for GFP (Fig 5A and 5B). A subpopulation of the GFP positive cells inside the tumor expressed NG2 (55 ± 12%) and CD13 (26 ± 15%), markers associated with activated pericytes and mesenchymal stromal cells (Fig 5C and 5D) [23]. Further on, the majority of the intratumoral GFP positive pericytes weakly expressed the pericyte marker α-SMA (86 ± 7.7%; Fig 5E). In these cells, α-SMA immunoreactivity was visualized as patches within the cytoplasm in close contact with the plasma membrane. All pericyte markers were represented among both morphologically different pericyte types, although CD13 seemed to be expressed predominantly on flat, elongated cells. A subset of the GFP positive pericytes labeled for Ki67 (16 ± 1.7%), indicating active proliferation (Fig 6A and 6B).

### Pericytes Do Not Label with Stromal Tumor Cell Markers or Inflammatory Cell Markers

**Fig 7:**
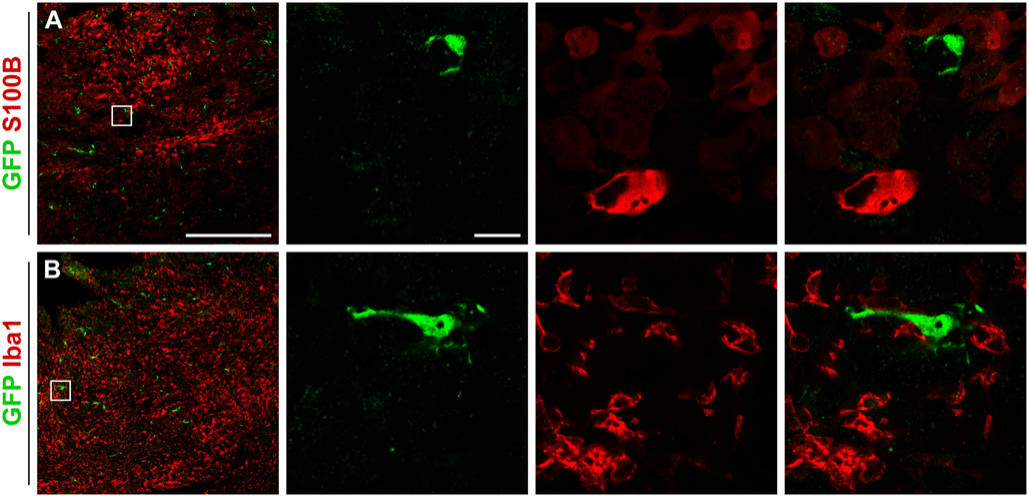
GFP positive cells become neither astrocytes nor microglia. None of the GFP positive cells express (A) S100B or (B) Iba1, ruling out the possibility that they become astrocytes or microglia. Scale bar is 500 μm in the low magnification images and 20 μm in the high magnification images.

Finally, we examined whether pericytes adopt a different phenotype within the tumor. GFP positive pericytes did not express the astrocyte marker S100B (Fig 7A) and all GFP positive pericytes within the tumors were negative for the microglia marker Iba1 (Fig 7B). However, Iba1 positive microglia cells were found in very close proximity to pericytes indicating a possible juxtracrine-like communication.

## Discussion

Here we provide evidence that the normal brain vasculature contributes the majority of pericytes to GL261 mouse glioma vasculature. Using a pericyte reporter mouse where activated pericytes express GFP [15], we show that the tumor vasculature of grafted glioma contains a high proportion of host-derived GFP positive cells. Furthermore, in response to unilateral growth of an intracranial tumor, a significant increase of activated pericytes was observed within the cortex of both the ipsilateral and contralateral hemisphere as well as in the ipsilateral SVZ, indicating a significant influence of local glioma growth on widespread areas of the mouse brain.

The present study uses the GL261 mouse glioma model. It carries both p53 and K-ras mutations, as does many of its human counterpart, GBM [17]. The model was chosen for the present study because it represents one of the very few mouse brain tumor models syngenic to the C57BL/6 mouse strain and it is widely used because of its well characterized similarities to GBM. In particular, the invasive and angiogenic properties of GL261 closely mimic that of human GBM [24].

The vast majority of pericytes in normal as well as pathological tissues, such as tumors, express PDGFR-β [25]. Interestingly, a majority of the PDGFR-β positive pericytes within the tumor were co-labeled for GFP, indicating that they are recruited from the host. This contrasts the recent work of Cheng et al. stating that a majority of the pericytes within GBMs are derived from the tumor itself [8]. In that study, *in vivo* cell lineage tracing demonstrated that glioma stem cells generate the majority of vascular pericytes in mouse and human GBM. The present study does not rule out the possibility that a proportion of pericytes within the tumor are derived from the glioma cells. Even though the GL261 mouse glioma model mainly consists of differentiated cells, it is a heterogeneous cell line containing subpopulations of cells with retained differentiation capacity [24,26,27]. In fact, the occurrence of GFP negative PDGFR-β positive pericytes, although at a considerably lower frequency than in [8], within mouse glioma would lend some support to such a clonal origin. Resolution of the discrepancy between the findings of the present study and that of Cheng et al. cannot be obtained at this stage, however, a differential capacity of tumor cell plasticity and differentiation potential between different models of glioma might be a contributing factor. Since we have used non-labeled tumor cells that cannot be traced, we are not able to draw any further conclusions about the origin of the GFP negative PDGFR-β positive pericytes. Furthermore, and importantly, whether brain pericytes are activated and recruited into other animal models of glioma and into highly malignant glioma in humans remains to be clarified.

Although many key molecular regulators of pericyte function and activation have been previously defined [28], the mechanism of widespread pericyte activation in response to local tumor growth at a considerable distance remains unknown. Principally, pericyte activation could result from the widespread parenchymal diffusion of factors produced locally by the tumor, from glioma-derived factors such as exosomes delivered by the systemic circulation [29] or the cerebrospinal fluid or, alternatively, from the action of elevated intracranial pressure and resulting hypoxia [30] or brain edema [31]. Interestingly, GFP positive pericytes were found in close vicinity of laminin positive tumor vessel microsatellites. This may indicate that pericyte activation and recruitment into glioma requires specific interaction with the laminin-rich vascular basement membrane of the GL261 tumor. However, in the present study, the activation of pericytes included structures also located in the contralateral hemisphere, at a considerable distance from the main tumor. The molecular basis for glioma-induced recruitment of pericytes from another distant site, the bone marrow, has been elucidated in some detail. Hypoxia-inducible factor-1α (HIF-1α), a direct mediator of tumor hypoxia, has been shown to mobilize and lead to tumor incorporation of bone marrow-derived vascular modulatory cells, including a small portion of pericyte progenitor cells [9]. This effect is mediated through the HIF-1 target stromal-derived factor-1α and recruitment is dependent on the presence of matrix metalloproteinase-9 and its ability to mobilize sequestered VEGF within the tumor. The importance of tumor hypoxia as a critical trigger of pericyte recruitment into glioma is further substantiated by the findings of large numbers of pericytes specifically in the penumbra around areas of hypoxia in the present paper.

Interestingly, also pericytes in the rostral SVZ were activated by tumor growth in the ipsilateral striatum. The SVZ is an active proliferative zone within the brain and this region has previously been shown to be reactive and produce nestin and doublecortin positive neuroblasts in response to glioma [32,33]. Furthermore, in response to local cerebral ischemia, precursors of pericytes within the SVZ proliferate and migrate to the infarcted area where they are incorporated into new vessels of the peri-infarct regions [34]. Whether pericyte activation in the SVZ facilitates this process remains to be established.

A portion of the GFP positive cells infiltrating the tumor were co-labeled for the proliferation marker Ki67, indicating that proliferation of endogenous brain pericyte precursor cells is actively involved in the process of glioma vascularization. Although proliferation is part of this process, the present study was not designed to clarify to what extent proliferation of perivascular progenitors contributes. Only a subset of the GFP positive cells within the tumor was co-labeled for Ki67. However, only actively proliferating cells at the exact time of tissue perfusion are labeled by this marker. Thus, these results might be an underestimation of the contribution of pericyte stem- or precursor cell proliferation as opposed to pericyte recruitment by the mechanism of tumor-tropic migration of existing, post-mitotic pericytes.

A subset of cells did not express NG2 showing that non-activated pericytes also reside within the tumor [23]. Furthermore, a majority of the GFP positive cells inside the tumor were negative for the pericyte marker CD13, also expressed by mesenchymal stromal cells. In contrast, GFP positive pericytes outside the tumor expressed CD13, thereby indicating a phenotypic shift as the cells enters the tumor. Interestingly, even though intratumoral RGS5 positive pericytes aligned close to cells expressing the pro-angiogenic factor VEGF-R, known to play a major role in glioma angiogenesis and possibly invasiveness [35], pericytes activated in response to glioma growth did not express VEGF-R themselves.

The intratumoral GFP positive pericytes were all positive for the pericyte marker PDGFR-β, but only a proportion of the cells expressed the pericyte markers NG2, CD13 and α-SMA. It is known that pericytes constitute a heterogeneous cell population with a marker expression that varies depending on the surrounding tissue [36]. Furthermore, the marker expression can be altered under pathological conditions. For example, α-SMA is upregulated on pericytes in the central nervous system in response to a tumor [37], and NG2 becomes upregulated in response to angiogenesis [23]. Thus, it is likely that intratumoral pericytes, possibly recruited at different time points to the tumor, do not share the same expression marker profile.

To exclude the possibility that the GFP positive cells within the tumor become astrocytes, they were stained for S100B that is expressed by mature astrocytes surrounding blood vessels [38]. A considerable amount of S100B positive cells were seen within the tumor, however none of the cells co-localized with GFP. We also investigated whether the recruited pericytes could become activated microglia, as we have recently shown in ischemic stroke [12]. However, although a large number of Iba1 positive microglia was present in the GL261 tumors, none of the cells co-expressed GFP indicating that the recruited pericytes do not become microglia within the mouse glioma tumor or peritumoral microenvironment.

Taken together, our findings show that pericytes become activated in widespread areas of the brain in response to GL261 mouse gliomas. Non-tumor-derived pericytes infiltrate the glioma extensively and integrate with the vasculature. The findings thus strongly support that this glioma model constitutes a mosaic of host-derived and tumor-derived cells rather than being predominantly of a single cell clonal origin. If these results are confirmed in human glioma, the findings may provide a rational basis for targeting pericyte activation in glioma therapy.
